# 

**Published:** 1887-08

**Authors:** 


### INDEX.

### A

PAGE.

Angell, Edward B., M. D. Some Suggestions Regarding the Management of Hysterical Disorders .......................... 1
Apostoli's Method of Electrolysis in the Treatment of Fibroid Tumors....... ............. 75
Acute Ichiopathic Pleuritis and Acute Articular Rheumatism, Analogy between ............ 77
Albuminuria in Health.......... 78
Ascites in Children............. 81
Accidental Vaccinia, A Case of, resulting in Death............ 86
Arseniate of Lithia in Diabetes.. 88
Aural Furuncles, Bacteriology of. By Dr. B. Lowenberg......... 102
Andrews, Judson B., M. D., Care of the Insane of the United States... .... .............. 104
American Gynecological Society. 140
Anæmia. By Frederick P. Henry, M D...................... 144
Acne, The Treatment of ....... 228
Anatomy, Descriptive and Surgical. By Henry Gray, F. R S 237
Antipyrin and Antifebrin Uses of......................... 276
Antidotes in Strychnine, Resorcin and Picrotoxine........... 278
Aitken, Wm., M. D Handbook of Treatment........ ... 287
Acid, Boracic, in the Treatment of Leucorrhœa............... 323
Anterior Nares, A Ready Method for Removing Foreign Bodies from the.................... 329
Acetanilide as a Nervine........ 331
Apomorphine................... 334

PAGE.

Acute Endocarditis, The Etiology of.................... .. ... 370
Antithermic Acetanilide as a Nerve Sedative............... 371
Aseptic and Antiseptic Surgery, Rules of By Arpad G. Gerster, M. D....... ............... 382
Abscess, Peri-Nephritic. By W. H. Heath, M. D.............. 385
Antipyrin in Migraine ......... 413
Antipyrin in Acute Bronchitis of Children.... ................ 415
Antipyrin. Poisoning by a Ten-grain Dose of ............... 417
Acetanilide, New Researches with................ ... 418
Antipyrin as a Uterine Sedative. 419
Alkaloid, a Toxic, constantly exhaled from the Lungs. .. .... 425
Atlas of Venereal and Skin Diseases. By Prince A Morrow, A. M, M. D....... .. 428 and 430
Attidella Reale Accademia Medica di Roma 1886–1887 ...... 429
Address to the Graduating Class of the Medical Department of Niagara University. By Henry Flood, M D.................. 433
Agnew, Death of Dr. C. R...... 464
Antipyrin in the Treatment of Seminal Emissions........... 470
Association, the American, of Obstetricians and Gynecologists.. 471
Asthma, Epileptic: By F. A. Harrington, A. M, M. D..... 496
Aphorisms, Medical............. 558
Antipyrin and Sweet Spirits of Nitre........................ 560
Advertisements, Quack, in the Religious Press. ............. 572

PAGE.

### B

Bacterial Research, The Fallacies of Popular. By George W. Lewis, A. M., M. D .......... 8
Burns, Treatment of. Lessons from the Richmond Fire. By George E. Fell, M. D., F. R. M. S....... ................. 55
Browne, Mr. Lennox. Treatment of Tuberculosis of the Larynx 115
Buffalo Law School............. 141
Butler, John S., M. D. The Curability of Insanity and the Individualized Treatment of the Insane ...................... 143
Bruen, Edward Tunis. Practical Lessons in Nursing........... 144
Buck, Albert H., M. D. A Reference Handbook of the Medical Sciences.................. 191
Bubo, Concerning the Treatment of, by Extirpation of the Glands. By W. H. Heath, M. D. ... ............ .......... 205
Buffalo Medical Library Association......................... 213
British Gynecological Society... 217
Buffalo Colleges.... ........... 230
Backus, Ogden, M.D., The Recognition of Incipient General Paresis ..................... 241
Barker, The Late Arthur M , M. D. ......................... 285
Bartholow, Roberts, M.A., M. D., LL. D. A Practical Treatise on Materia Medica and Therapeutics......................... 288
Bladder, For Washing out the... 310
Burns and Frostbites, A New Treatment for............. .. 372
Buffalo State Asylum for the Insane ...................... 373
Bladder, a Case of Bursting of the Bladder, with Remarks. By Dr. Marcell Hartwig...... 393
Bedbugs, Fleas and Lice, The Use of.................... 401
Benzoate of Sodium and Uremia 418

PAGE.

Buffalo Medical College ....... ... 420
Buffalo Obstetrical Society, Transactions of the.... ........... 459
Buffalo Obstetrical Society...... 476
Bailey, Wm. C., M. D. An Inquiry into the Importance of Physical Culture as a Branch of the Science of Prevention.. 481
Benzoate of Sodium in Throat Affections .................. 506
Brubaker, Arthur, A. M., M.D. A Compend of Human Physiology 528
Bard, Dr. L. On Leucocythæmia, considered as Specific Cancer of the Blood................ 561

### C

Chemical Bodies, Immunity by Injection of.................. 34
Cynophobia.................... 42
Cancer in Japan.. ............ 83
Corrosive Sublimate Poisoning after Injections of.......... .... 86
Cocaine Inebriety .. .......... 87
Contractions of the Uterus throughout Pregnancy. By J. Braxton Hicks, M. D., F. R. S. 97
Cervix Uteri. Rapid Dilatation of the. By Dr. W. W. Wathen.. 112
Congress, What Prominent Men say of the.................. 124
Constipation, Infantile.... .... 177
Correction..................... 189
Cutler, C. W., M. S., M. D. Differential Diagnosis of Diseases of the Skin.................. 192
Clinical Diagnosis, Manual of. By Dr. Otto Seifert and Dr. F. Mueller...................... 192
Clark, Edward, M. D. A Study of some of the Rectal Disorders of Pregnant Women.......... 193
Cocaine, A Curious Effect of. By Frank Hamilton Potter, M. D.. 210
Colles Fracture, A Contribution to the Study of. By D. M. Totman, M. D............... 212
Chorea, One Cause of.......... 229

PAGE.
Confinement, The Hour of...... 230
Cancer, Vaginal Hysterectomy for.......................... 232
Chapman, Henry C., M. D. Treatise on Human Physiology, for the use of Students and Practitioners of Medicine........... 239
Corns, Treatment of ........... 271
Carcinoma of the Uterus........ 274
Constipation, Another Cure for.. 311
Contempt of Court............. 319
Cancer and Cancerous Diseases. 321
Compound Salicylated Plaster, On the Advantages of a....... 330
Canadol, A new local Anæsthetic 332
Cold on the Heart. By F. R. Campbell, M. D.............. 359
Cascara Sagrada, Tasteless Preparations of.................. 366
Concentration of the Blood as a Cause of Convulsions......... 368
Cancer of the Cervix........... 420
Catarrhe Cronique Hypertrophique et Atrophique des Fosses Nasales, etc. By Garrigou-Desarenes.................... 430
Clark, Alonzo, M. D., LL. D. Lectures on Diseases of the Heart........................ 432
Cronyn, John, M. D. Address on Medicine. ...... ......... 441
Constipation, Habitual.......... 469
Campbell, F. R., A. M., M. D. The Language of Medicine ... 526
Consumption Contagious?...... 559
Cascara Sagrada in Rheumatism. By H. T. Goodwin, M. D..... 566

D

Diarrhœa, Treatment of, by Iodoform and Charcoal....... 17
Drug Eruptions. By Prince A. Morrow, A. M., M. D. ....... 48
Diseases of the Hair and Scalp, A Practical Treatise on the. By George Thomas Jackson, M. D. 96
Drugs, Certain, on the Circulation of the Kidneys. By Dr. Charles D. F. Phillips........ 106

PAGE.
Deniau, Dr. L. Strophanthus Hispidus.................. 165
Diabetes, A Convenient Formula for the Treatment of, by Lithium in Pill Form......... 172
Department of Health of Buffalo, Annual Report of the.......... 185
Diphtheria, A Treatise on. By Dr. A. Samie................ 190
Diseases, Nervous, and their Diagnosis. By H. C. Wood, M. D., LL. D ................ 190
Diseases of the Skin, Differential Diagnosis of the. By C. W. Cutler, M S., M. D. ..... 192
Duodenum, A New Theory in Regard to the Function of the. 230
Disease, that Terrible, The Divers 263
Doctors in Russia........ ..... 265
Dosimetric Therapeutics as Applied to Pneumonia and Bronchitis.... .......... ...... 266
Diarrhœa, Infantile............ 318
Diphtheria ................. ... 363
Doctor and Patient. By S Weir Mitchell, M. D , LL.D......... 383
Drugs of New York............ 417
Diseases of the Skin, A Practical Treatise on. By John V. Shoemaker, M.D. ........... 431
Diseases of the Heart, Lectures on. By Alonzo Clark, M. D., LL.D. ....... ............ 432
Diagnosis, The, of Contracted or Granular Kidney. By A. D. Lake, M. D.................. 447
Diseases of the Genito-Urinary Organs, The Surgical. By E. L. Keyes, M. D ........... 419
Diseases, The Infectious. By Prof. Karl Liebermeister...... 575

E

Eliot, Gustavus, A. M., M. D. The Treatment of Neuralgia in General Practice ........... 157
Expert, An. .......... .... 265
Epistaxis, Treatment of......... 272

PAGE.

Ergotine, On the Subcutaneous Use of, in Diabetes and Albuminuria........................ 280
Experiments bearing on the Infliction of the Death Penalty by Electricity...... ........ 303
Examination for License to Practice........................ 311
Enemata, Nutrient........ .... 312
Elsner, H. L. Early Diagnosis of Tubercular Meningitis in Children... ............... .... 337
Excessive Corpulence, The Guiding Principles of Treatment in. 409
Erythrophlœin, Notes on Another Supposed Local Anæsthetic. By Alvin A. Hubbell, M. D. 458
Electricity, A Practical Treatise on the Medical and Surgical Uses of. By George M Beard, A. M., M. D., and A. D. Rockwell, A. M., M. D............ 478
Essentials of Physiolcgy, Questions and Answers on the. By H. A. Hare, M. D........ ... 480
Essentials of Chemistry and Toxicology. By A. R. Witthaus, A. M., M. D............ 480
Electro-Magnet in Removal of Steel from the Interior of the Eye. By Alvin A. Hubbell, M. D...................... 545
Essentials of Medical Chemistry and Urinalysis. By S. E. Woody, A. M., M. D......... 576

F

Fibroids of the Uterus, On the Treatment of, by Electricity. By W. Windham Webb, M. D., M. R. C. P., London......... 66
Follicular Tonsilitis and Idiophatic Peritonitis............. 182
Fell, Geo. E., M D., F. R. M. S. Treatment of Burns. Lessons from the Richmond Fire...... 55

PAGE.

Fell, George E., M. D., F. R. M. S. Forced Respiration in Opium Poisoning, its Possibilities, and the Apparatus best adapted to produce it.......... 145
Fever, Typhcid................ 270
Fever, The Influence of upon Insanity.................... 275
Fractures, Treatment of, by Massage. By F. A. Harrington, M. D.................... .. 308
Free Trade for Physicians....... 333
Fell, Geo. E , M. D., F. R. M. S. Forced Respiration, Case No. 3. 345
Frederick, C. C , M. D. Hystero-Epilepsy complicating Pregnancy...................... 353
Fox, Dr. George H. Photographic Illustrations of Skin Diseases.................. .. 383
Fever Nursing, Practical Lessons in. By J. C. Wilson, A. M., M. D........................ 384
Fevers, Periods of Isolation for Eruptive.................... 400
Fever, Chagres, at Panama..... 403
Flood, Henry, M. D. Address to the Graduating Class of the Medical Department of Niagara University.................. 433

G

Gonorrhœa Injection for........ 20
Gastric Ulcer, Pathogeny of .... 36
Gaseous Enemata............. 37
Greene, Walter D., M. D. Pelvic Cellulitis ................ 49
Guaiac in Quinsy............... 64
Gaseous........................ 66
Gonorrhœa and Spermatorrhœa, On the Pathology and Treatment of. By J. L. Milton.. .. 96
Gray's, A New Edition of....... 171
Gynecology, A System of, by American Authors. By Mathew D. Mann, M. D...... ...... 189

PAGE.

Granger, Wm. D., M. D. Increase of the Insane, and some Problems connected with their Public Care .................. 220
Gray, Henry, F. R. S. Anatomy, Descriptive and Surgical....... 237
Gynecology, Lessons in. By Wm. Goodell, A. M., M. D... 240
Goodell, Wm., A. M., M. D., Lessons in Gynecology. ..... 240
Gonorrhœa and Sterility........ 265
Gastrotomy, A Second, for the Removal of a Foreign Body from the Œsophagus.......... 273
Gastric Ulcer, The Etiology of.. 331
Garmany, J. J., A. M., M. D. Operative Surgery on the Cadaver.......................... 336
Gerster, Arpad G., M. D. Rules of Aseptic and Antiseptic Surgery ............ .......... 382
Graduates in Medicine in the United States ......... ....... 416
Garrigou—Desarene. Du Catarrhe Chronique Hypertrophique et Atrophique des Fosses Nasales. etc.................. 430
Goodwin, H. F., M. D. Cascara Sagrada in Rheumatism...... 566

H

Hysterical Disorders, Some Suggestions regarding the Management of. By Edward B. Angell, M. D................ 1
Hypnotic Medication........... 85
Hicks, J. Braxton, M. D., F. R. S. Contractions of the Uterus throughout Pregnancy........ 97
Henry, Frederick P., M. D. Anæmia..................... 144
Heath, W. H., M. D. Concerning the Treatment of Bubo by Extirpation of the Glands...... 205
Howe, Dr. Lucien.............. 235
Harrington, Frank A., M. D. On the Subcutaneous Injection of Blood, Infusion (subcutaneous) and Intravenous Transfusion.. 258

PAGE.

Hæmorrhoids, Treatment of Internal, by Injections.......... 270
Hamilton, Allen McLane, M. D. Hamilton's Medical Jurisprudence......... ............. 286
Hand-book of Treatment. By Wm. Aitken, M. D........... 287
Hayd, Herman E., M. D., M. R. C. S Treatment of Chronic Stomach Troubles by Irrigation 289
Harrington, F. A., M. D. Treatment of Fractures by Massage. 308
Hymen, The Nature of the...... 316
Hypnotic, A New............... 332
Health Physician, The.......... 332
Hystero-Epilepsy complicating Pregnancy. By C. C. Frederick, M. D....... ................ 353
Heart and Lungs, Contributions to the Study of the. By James R. Leaming, M. D............ 384
Heath, W. H., M. D. Peri-Nephritic Abscess....... .... 385
Hartwig, Dr. Marcell. A Case of Bursting of the Bladder, with Remarks............... 393
Hydrastis Canadensis, Contribution to the Study of........... 413
Hemorrhage, Uterine........... 444
Hygiene, The, of the Skin, or the Art of Preventing Skin Diseases. By A. Ravogli, M. D.. 431
Hubbell, Alvin A., M. D. Note on Erythrophlœin, another supposed local Anæsthetic. ... 458
Hare, H. A., M. D. Questions and Answers on the Essentials of Physiology................ 480
Harrington, F. A., A. M., M. D. Epileptic Asthma............. 496
Hysteria and Brain Tumor. By Mary Putnam Jacobi, M. D... 527
Hubbell, Alvin A., M. D. The Electro-Magnet in the Removal of Steel from the Interior of the Eye..................... 545
Hewitt, Grailey, M. D. The Pathology, Diagnosis, and Treatment of the Diseases of Women. 575

PAGE.

### I

Iodoform, Modes of Disguising the Odor of .............. 26
Iodol: An Effective Substitute for Iodoform.................... 84
Insane, Care of the, in the United States. By Judson B. Andrews, M. D....................... 104
Inducement, An.................. 173
Insane, Increase of the, and some Problems connected with their Public Care. By Wm. D. Granger, M. D............... 220
Incipient General Paresis, The Recognition of. By Ogden Backus, M.D................ 241
Injection, On the Subcutaneous, of Blood. Infusion (subcutaneous) and Intravenous Transfusion. By Frank A. Harrington, M. D................... 258
Interstinal Affections, Antiseptic Treatment of................. 263
Inquiry, An. into the Importance of Physical Culture as a Branch of the Science of Prevention. By Wm. C. Bailey, M. D...... 481
Intubation of the Larynx, A German View of ................ 505
Intra-Uterine Tamponing....... 508
Inflammations, Remarks on Pelvic, and the Management of their Residues. By Wm. W. Potter, M. D................ 530
Infant Feeding, Art of. By W. I. Thayer, D. D. S., M. D., Brooklyn, N. Y.............. 554
Inocculation, The Method of, in Brazil and Mexico............ 559

### J

Jackson, George Thomas, M. D. A Practical Treatise on the Diseases of the Hair and Scalp, 96
Jenks Memorial Prize, The Wm. F...................... ..... 379

PAGE.

Jacobi, Mary Putnam, M. D. Hysteria and Brain Tumors... 527
Jenks, Edward W., M. D. The Disorders of Menstruation.... 576

### K

Keys, E. L., A. M., M. D. The Surgical Diseases of the Genito-Urinary Organs.............. 479

### L

Lewis, George W., Jr., A. M., M. D., The Fallacies of Popular Bacterial Research............ 8
Leonard, C. Henry, The Vest-Pocket Anatomist............ 48
Lutand, Dr. M. Pasteur et la Rage........................ 95
Labor, Mechanism of. By Prof. T. Lazarewitch, M. D......... 101
Lazarewitch, Prof. T., M. D. Mechanism of Labor......... 101
Lowenberg, Dr. B. Bacteriology of Aural Furuncles............ 102
Larynx Intubarion of the. By Dr. Joseph O'Dwyer.......... 108
Lupus Erythematosus. By Dr. Ravogli.......................... 119
Leprosy Scare, The............. 283
Laparotomy in Suppurative and Tubercular Peritonitis ....... 324
Lavage in Children............. 373
Leaming, James R., M D. Contributions to the Study of the Heart and Lungs............. 384
Lake, A. D., M. D., Perrysburg, N. Y. The Diagnosis of Contracted or Granular Kidney.... 447
Lung, Inundation of the........ 464
Leucocythæmia, Considered as Specific Cancer of the Blood. By L. Bard, M. D............ 561
Liebermeister, Prof. Karl. The Infectious Diseases........... 575

### M

Medical Logic ................ 19

PAGE.
Medical Examiners, The New York State Board of.......... 29
Microbe of Cancer, The ........ 33
Morrow, Prince A., A. M., M. D., Drug Eruptions............... 48
Morse, Willard, H., M. D. A Word as to Papoid............ 63
Mamma in an Ovarian Tumor... 65
Medical School, Selection of..... 88
Medical Law, Codified, of New New York State.............. 21
Medical Congress, International.. 91
Milton, J. L On the Pathology and Treatment of Gonorrhœa and Spermatorrhœa........... 96
Menstruation, Vicarious. By Duncan C. McCallum, M. D., M. R. C. S......................... 99
McCallum, Duncan C., M. D., M. R. C. S. Vicarious Menstruation.......................... 99
Medical Congress, Notes and Gossip about the International ... 128
Medical Education in America... 137
Medical Congress, The Ninth International. ....... . ..... 174
Mann, Matthew D., M. D. A System of Gynecology by American Authors ........... 189
Medical Sciences, A Reference Handbook of the. By Albert H. Buck, M. D.............. 191
Medicines The Therapeutic Value of Some, in the Treatment of Hemorrhagic Conditions of the Uterus. By C. D. Palmer, M. D...................... 201
Medical Association, The New York State.................. 236
Materia Medica, A Manual of Organic. By John M. Maisch, Ph. D...................... 240
Maisch, John M., Ph. D. A Manual of Organic Materia Medica..................... 240
Medical Society, Cattaraugus County.................... .. 262

PAGE.
Medical Jurisprudence, Hamilton's. By Allen McLane Hamilton, M. D.................. 286
Materia Medica and Therapeutics, A Practical Treatise on. By Robert Bartholow, M. A., M. D., LL. D.................. 288
Microscopy, Practical, A Course of Normal Histology for Students and Practitioners of Medicine. By Maurice N. Miller, M. D ...................... 335
Miller, Maurice N., M. D., Practical Microscopy, A Course of Normal Histology for Students and Practitioners of Medicine. 335
Materia Medica An Index of. By Chas. A. May, M D ......... 336
May, Chas. A., M. D. An Index of Materia Medica............ 336
Medical Education, Promoting the Cause of Higher............. 381
Mitchell, S. Weir, M. D., LL. D. Doctor and Patient........... 383
Medicines, Physiological Action of. By Louis Starr, M. D., James B. Walker, M. D., and W. M. Powell, M. D.......... 384
Morrow, Prince A., A. M., M. D. Atlas of Venereal and Skin Diseases............. 428 and 480
Medicine, Address on. By John Cronyn, M. D ............... 441
Memoriam, In.................. 516
Medical Nomenclature, A Revised .... ... ............... 522
Medical Association, The Cincinnati Meeting of the American.. ...................... 524
Medicine, The Language of. By F. R. Campbell, A. M., M. D.. 526
Medicines in Germany, The Suppression of Quack............ 560
Memoriam, In, The late A. R. Davidson, M. D ............. 568
Medical Department of Niagara University............... ... 569

PAGE.
Menstruation, The Disorders of. By E. W. Jenks, M. D........ 576
Medical Association, Transactions of the New York State, for the Year 1887............. 576

### N

Nerves, Trophic................ 38
Njimo Wood.................... 40
Nasal Vertigo.................. 80
Nymphomania, Etiology and Diagnosis of. By C. H. F. Routh, M. D....................... 130
Novel Departure in Advertising. 143
Nursing, Pract cal Lessons in. By Edward Tunis Bruen......... 144
Neuralgia, The Treatment of, in General Practice. By Gustavus Eliot, A. M., M. D........... 157
Nitro-Glycerine, Indications for the Use of................... 226
Nomenclature, Personal......... 307
Naphthol as an Antiseptic Medicament .................... 317
Niagara University, Annual Convocation of............... ... 425
Niagara University............. 472
Niagara University, Annual Meeting of the Alumni Association, Medical Department of....... 475
Naupathia.... ................. 563

### O

O'Dwyer. Dr. Joseph. Intubation of the Larynx............ 108
Opium Poisoning, Forced Respiration in. Its possibilities and the apparatus best adapted to produce it. By Geo. E. Fell, M. D., F. R. M. S.... ....... 145
Obstetric Synopsis. By John S. Stewart, M. D................ 432
Olive Branch in American Politics......................... 520
Œdema of the Lungs, A Case of. 560
O'Brien's, Dr. Nomination for the Position of Health Officer of the Port of New York..... 571

### P

PAGE.
Pasteur's Method............... 16
Pityriasis, Versicolor. ......... 19
Peritonitis, Experimental ....... 40
Providence Asylum for the Insane and Inebriate ........ 46
Pelvic Cellulitis. By Walter D. Greene, M. D................ 49
Papoid, A Word as to. By Willard H. Morse, M. D.......... 63
Premature Labor, Indications for the Induction of.............. 71
Phthisis, Analine Treatment of.. 87
Pasteur et la Rage. Par Dr. Lutaud... ..................... 95
Pathological Anatomy and Pathogenesis, A Text-book of. By Ernst Ziegler ................ 95
Phillips, Dr. Charles D. F. Certain Drugs on the Circulation of the Kidneys... .... ...... 106
Puerperal Fever, The Prevention of .......................... 181
Proceedings of the American Society of Microscopists...... 191
Physicians' Visiting List for 1888, 192
Palmer, C. D., M. D. The Therapeutic Value of some Medicines in the Treatment of Hemorrhagic Conditions of the Uterus ...................... 201
Potter, Frank Hamilton, M. D. A Curious Effect of Cocaine .. 210
Physiology, Treatise on Human, for the use of Students and Practitioners of Medicine. By Henry C. Chapman, M. D ... 239
Potter, Frank Hamilton, M. D. Tuberculosis of the Nose, Mouth and Larynx........... 295
Placenta, Management of the, in the Third or Placental Stage of Labor ........... ......... 313
Prostatic Calculus, A Case of. By J. W. Philpott, M. D...... 164
Philpott, J. W., M. D. A Case of Prostatic Calculus......... 164

PAGE.

Peritoneal Adhesion, The Diagnosis and Separation of, around the Displaced Uterus and Ovaries.......................... 327
Potter, Frank Hamilton, M. D. A Nasal Scissors.............. 358
Pharmaceutical Substitution..... 364
Physician, The Observing....... 412
Potter, Fr. H., M. D. Notes on the Treatment of Acute Tonsilitis in Children......... .... 455
Pathology, The Relation of, to the Practice of Medicine and Surgery. By F. W. Ross, M. D.. 489
Physiology, A Compend of Human. By Arthur Brubaker, A. M., M. D................ ... 528
Potter, William Warren, M. D. Remarks on Pelvic Inflammaations and the Management of their Residues................ 530
Pharmacy in the Sixteenth Century......................... 559
Phenacetine, The New Antipyretic......................... 574
Pathology, Diagnosis and Treatment of the Diseases of Women. By Grailey Hewitt, M. D....................... 575
Physician's Leisure Library..... 575

### Q

Quinine, To Give............... 173

### R

Respiratory Organs, The Local Treatment of Diseases of the.. 39
Rochester's, Dr., Successor...... 41
Remedies, New, McKesson & Robbins......... .......... 94
Ravogli, Dr. Lupus Erythematosus........ ................ .. 119
Routh, C. H. F., M. D. Etiology and Diagnosis of Nymphomania........................ 130
Rectal Disorders, A Study of some of the, of Pregnant Women. By Edward Clark, M. D........... .......... 193

PAGE.

Rochester Pathological Society.. 260
Resorcin...................... 277
Respiration, Forced. Case No. 3. By Geo. E. Fell, M. D., F. R. M. S......................... 345
Remedy for Croup, A Chinese... 363
Remedy for Phthisis, A Recent.. 372
Respiration, Artificial. Fell's Method........... ......... 375
Respiration, Forced, without Tracheotomy.................... 401
Ravogli, A., M. D. The Hygiene of the Skin or the Art of Preventing Skin Diseases......... 431
Reflex Neurosis due to Constipation.......... ............. 471
Ross, Frank D,, M. D. The Relation of Pathology to the Practice of Medicine and Surgery.. 489

### S

Students, Number of, in German Universities......... ....... 15
Stockton, Dr., Successor to . ... 45
Skin Disease................... 66
Succus Alterans in Rheumatism and Syphilis... ............ .. 88
Strophanthus Hispidus. By Dr. L. Deniau.................... 165
Scarlet Fever, Preventive Inoculations for..................... 169
Stenocarpine Sensation. An Attempt to Impose upon the Medical Profession............... 170
Syphilis, The Treatment of, by Hypodermic Injection........ 179
Supra-Vaginal Hysterectomy, How shall we Manage the Pedicle in....................... 183
Samie, Dr. A. A Treatise on Diphtheria.................. 190
Sublimate Paper as a Surgical Dressing ................... 278
Stomach Troubles, Treatment of Chronic, by Irrigation. By Herman E. Hayd, M. D., M. R. C. S...................... 289
Septic Intoxication, Contribution to the Study of............... 319

PAGE.
Syphilis, The Treatment of...... 328
Surgery, Operative, on the Cadaver. By J. J. Garmany, A. M,, M. D....... ........ 336
Scissors, A Nasal. By Frank Hamilton Potter, M. D........ 358
Surgery........... ........... 380
Skin Diseases, Photographic Illustrations of. By Dr. Geo. H. Fox............... ........ 383
Student Life in Switzerland...... 403
Sterility in the Male............ 411
Shoemaker, John V. A Treatise on Diseases of the Skin. ..... 431
Stewart, John S., M. D. Obstetric Synopsis.................. 432
Syphilis of the Endometrium.... 466
Strophanthus, The Action of.... 469
Stone in the Bladder, A Strange Case of.................... 502
Snake Bite and its Antidote...... 560

T

Tablet Triturates............... 17
Tubercular Phthisis, The Topical Treatment of................ 35
Tobacco, Effects of, upon the Health............. ..... 65
Thyroid Gland, Function of the. 82
Tuberculosis, Treatment of, of the Larynx. By Mr. Lennox Browne.... ..... ......... 115
Totman, D. M., M. D. A Contribution to the Study of Colles Fracture....................... 212
Tyrotoxicon, An Interesting Case of Poisoning by.............. 222
Tape-Worm in Denmark........ 225
Tape-Worms, Ninety, in one Girl........................ 264
Tobacco, The Deleterious Effects of, on the Throat and Nose... 277
Tobacco, The Action of...... ... 279
Tuberculosis of the Nose, Mouth and Larynx. By Frank Hamilton Potter, M. D ........... 295
Telephone, The Wonders of the. 311
Tubercular Meningitis in Children, Early Diagnosis of. By H. L. Elsner................. 337
Tape-Worm................... 364
Tonsilitis in Children, Notes on the Treatment of Acute. By Frank Hamilton Potter, M. D.. 455

PAGE.
Thayer, W. J., D. D. S., M. D., Brooklyn, N. Y. Art of Infant Feeding..................... 554
Tetanus, Two Cases of........ .. 560

U

Urethral Stricture, The Treatment of, by Electrolysis....... 224
Uterus and Morphinomania...... 227
Uterine Appendages, Their Removal by Abdominal Section... 281
Uterine Disorders, Sexual Relations as Causes of............ 320

V

Vest-Pocket Anatomist, The. By C. Henri Leonard..... ...... 48
Very Suggestive and Promising. 228
Van Duyn, Dr. J. Gun-shot Wound of the Chest.......... 251
Von Brücke, Honors to.......... 560

W

Webb, W. Windham, M. D., M. R. C. P., London. On the Treatment of Fibroids of the Uterus by Electricity.......... 66
Wathen, Dr. W. H. Rapid Dilatation of the Cervix Uteri..... 112
Whooping Cough, Newest Treatment for....... .......... . 172
Wood, H. C., M. D., LL. D. Nervous Diseases and their Diagnosis.................... 190
Wound, Gun-shot, of the Chest. By Dr. J. Van Duyn.......... 251
Wounds, The Treatment of, by Iodoform Tampons........... 325
Water, The Use of, at Meals ... 369
Waters of New York Harbor.... 376
Wilson, J. C., A. M., M. D. Practical Lessons in Fever Nursing. 384
Whooping Cough, The Bacillus of.... ..................... 406
Witthaus, R. A., A. M., M. D. Essentials of Chemistry and Toxicology................... 480
Woody, S. E., A. M., M. D. The Essentials of Medical Chemistry and Urinalysis............ 576

Z

Ziegler, Ernst, A Text-book of Pathological Anatomy and Pathogenesis................ 95

